# Postoperative Cognitive Dysfunction in the Elderly: A Role for Modafinil

**DOI:** 10.7759/cureus.26204

**Published:** 2022-06-22

**Authors:** Ronak Desai, Kinjal Patel, Sandeep Krishnan, Ludmil V Mitrev, Keyur Trivedi, Marc Torjman, Michael Goldberg

**Affiliations:** 1 Anesthesiology, Cooper University Hospital, Camden, USA; 2 Anesthesiology, Wayne State University School of Medicine, Detroit, USA; 3 Anesthesiology, Thomas Jefferson University, Philadelphia, USA; 4 Anesthesiology, University of Miami Miller School of Medicine, Jackson Memorial Hospital, Miami, USA

**Keywords:** neurocognitive, surgery, dysfunction, cognitive, modafinil

## Abstract

Introductionː Postoperative cognitive dysfunction has long-term consequences of increased mortality, loss of autonomy, and prolonged hospitalization. We sought to determine whether exposing patients to modafinil may attenuate or prevent this devastating syndrome from affecting the elderly postoperatively.

Methodsː Adults aged 65 and older and American Society of Anesthesiologists (ASA) I-III physical status scheduled for elective noncardiac/non-neurosurgical surgery were included. Subjects were tested with the Trail Making Test (TMT) and Rey Auditory Visual Learning Test (RAVLT) preoperatively as well as in the immediate postoperative period, at 1 week, and at 3 months. After baseline testing, patients were randomized into three groups: 0) placebo pre and post-procedure; 1) modafinil only pre-procedure and placebo post-procedure; and 2) modafinil pre and post-procedure. A nonsurgical control group was also utilized.

Resultsː Seventy-six subjects completed the trial 3 months post-surgery. The baseline RAVLT obtained was analyzed with 2-way ANOVA with repeated measures and showed improvement in learning in all groups (p = 0.03). At 1-week post-surgery, Group 0 subjects demonstrated no learning improvement in the RAVLT. However, there was a significant improvement in learning in both groups that received modafinil (p<0.01), and in the nonsurgical controls (p<0.01). This learning benefit normalized at 3 months.

Conclusionː In this prospective, double-blind, placebo-controlled trial, we found that patients who received modafinil showed improvement in the RAVLT at 1 week. However, this learning benefit normalized at 3 months. Further study should examine dose effect, timing, and route of administration to determine if the effect can be enhanced and if in fact, wakefulness is improved post-surgically.

## Introduction

Postoperative cognitive dysfunction (POCD) is a common complication among the elderly after major surgery. Recent studies have estimated the prevalence of POCD to be 41% at hospital discharge and as high as 13% at 3 months postoperatively in the elderly. The unknown is the etiology of this cognitive decline, but age has been thought to be an important risk factor, and with an aging population, the number of elderly patients undergoing surgery is likely to grow in the future [1]. Another potential predictor of POCD is the presence of preoperative cognitive impairment. Silbert and colleagues demonstrated a greater incidence of POCD at 7 days and 3 months in those patients undergoing hip arthroplasty who had preexisting cognitive impairment [2].

A recent review of the literature demonstrates a consensus that exposure to general anesthesia can speed cognitive decline in the elderly population [3]. Furthermore, there is strong evidence from animal experiments to suggest that standard doses of routine anesthetics may produce long-lasting learning and memory impairments that persist for weeks or months after anesthetic exposure [4-6].

Our research group previously demonstrated that young patients who underwent outpatient procedures with general anesthesia felt fatigued and drained. These same patients were able to have their symptoms reversed with the use of modafinil (2-(diphenylmethyl) sulphinyl acetamide) [7]. The effectiveness of modafinil in reducing excessive sleepiness has been examined extensively in clinical trials for the following sleep disorders; narcolepsy, obstructive sleep apnea/hypopnea syndrome (OSAHS), and shift work sleep disorder (SWSD). The symptoms of certain psychiatric disorders like, major depression, schizophrenia, Parkinson’s disease, and attention-deficit hyperactivity disorder may be relieved by modafinil as well [8-11]. The compound is also used off-label by college students to enhance their ability to concentrate.

To date, large-scale prospective clinical protocols looking at ways to minimize postoperative neurocognitive decline are lacking and so we designed a prospective, double-blind, placebo-controlled (and controlled intervention) protocol to determine if modafinil would prevent or blunt the decline in cognitive function in an elderly population undergoing noncardiac surgery. We further utilized a nonsurgical control (NSC) group to determine whether the effects of surgery alone contributed to POCD.

## Materials and methods

Patients were recruited for participation from January 2008 to January 2013 at Cooper University Hospital (CUH) after CUH institutional review board approval (IRB #08-086) was obtained. Registration was completed at ClinicalTrials.gov (#NCT03914118). Adult male and female subjects aged 65 and older and American Society of Anesthesiologists (ASA) I-III status scheduled for elective noncardiac and non-neurosurgical surgery requiring general anesthesia lasting greater than two hours were included in and consented to the study. Exclusion criteria are listed in Table 1. Consented subjects were tested with a Trail Making Test (TMT) and Rey Auditory Visual Learning Test (RAVLT) preoperatively as well as in the immediate postoperative period, at 1 week, and at 3 months. These time intervals were selected to avoid any contribution of the acute effects of surgery and to aid our understanding of neurocognitive deterioration over time in this elderly population. A Verbal Wakefulness Score was also utilized to ascertain wakefulness and recovery of cognition for this study. The effect of the level of education was examined.

**Table 1:** Exclusion Criteria CO_2_: carbon dioxide; BMI: body mass index

Criteria
Narcotic or illicit drug abuse
Chronic opioid use
Chronic use of anxiolytics
History of pulmonary disease
Co_2_ retention
Known sleep apnea
Obesity (BMI>30)
Known allergy to modafinil
History of stroke
History of neurological disease affecting cognition
History of severe cardiac disease
History of liver disease
History of renal insufficiency or failure

After baseline preoperative testing, patients were randomized into three groups, utilizing a 1:1:1 scheme. The three groups were: 0) placebo taken orally pre- and post-procedure (n = 20); 1) modafinil 200 mg taken orally pre-procedure and placebo post-procedure (n = 30); and 2) modafinil 200 mg taken orally pre and immediately post-procedure (n = 26).

The anesthetic was standardized by utilizing up to 1.2 minimum alveolar concentration (MAC) end-tidal concentration of isoflurane for maintenance. Opioids were administered routinely, but benzodiazepines, metoclopramide, and dexamethasone were avoided. Surgeries requiring nasogastric tube or orogastric tube placement were also excluded. Postoperatively, patients received standard pain treatment protocols and were weaned to oral medications as soon as they could tolerate a liquid diet. All patients were discharged from the hospital on standard doses of oral opiates as needed for pain control. Any patient that required an additional operation prior to their 3-month cognitive test was excluded from the analysis.

Nonsurgical controls

The effect of surgery and anesthesia alone on POCD was examined through the addition of a NSC group (Group 3). This group, which did not have surgery or receive study medication, took the same cognitive tests at the same time intervals as the placebo and treatment groups, with the exception of the immediate postoperative testing.

Previous studies have demonstrated that repeated neuropsychological testing may result in an improvement in test performance, or a practice effect [12-13]. Therefore, all study data were revised to include these 38 NSCs consented after completion of the three groups of patients randomized according to the originally approved protocol. These 38 subjects were screened and consented to in a physician’s outpatient office, or in the hospital, after meeting the age and health status inclusion criteria. Some may consider this sample to be a convenience sample; it does not exactly fit the definition since we used a set of inclusion/exclusion criteria. A total of three tests were performed on those subjects (baseline, 1 week, and 3 months). All 1-week and 3-month follow-up visits took place in the subject’s home which was identical to the surgical patients.

Neuropsychological assessment

**Figure 1:**
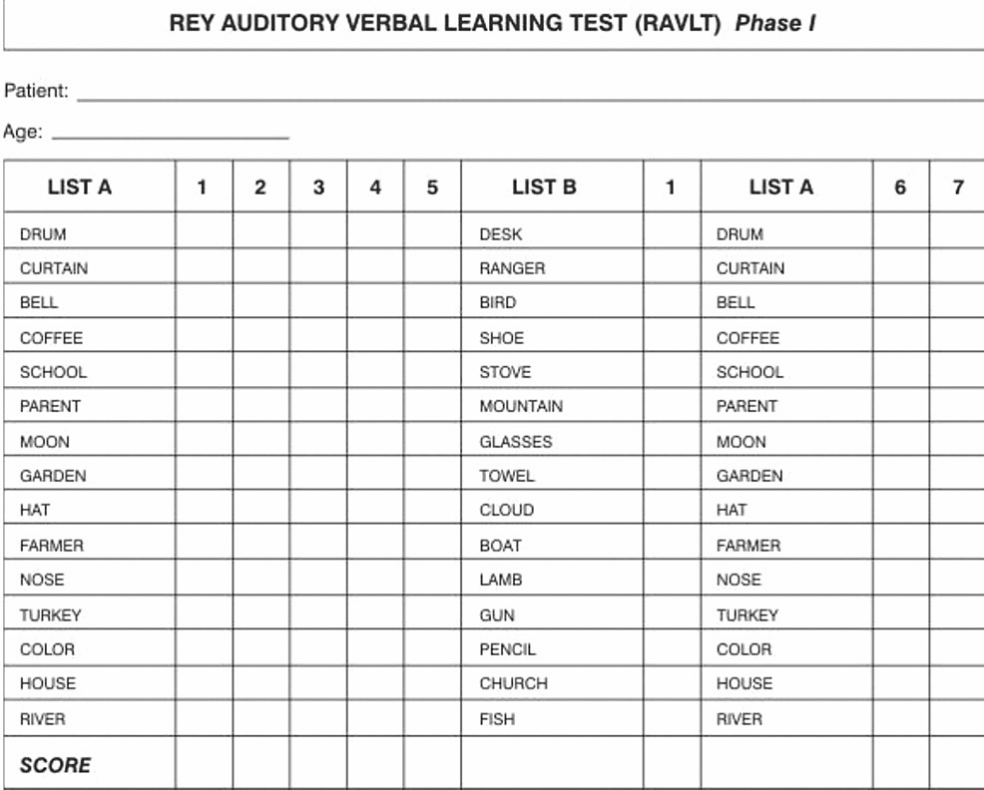
Rey Auditory Verbal Learning Test (RAVLT)

The RAVLT (Figure 1) evaluates a wide diversity of functions in both children and adults: short-term auditory-verbal memory, rate of learning, learning strategies, retroactive, and proactive interference, presence of confabulation or confusion in memory processes, retention of information, and differences between learning and retrieval [14-16]. Better recollection of words from the beginning of the list (primacy) reflects stronger, more active learning while the recollection of words from the end of the list (recency) reveals more passive learning [17]. The RAVLT has been shown to have a high test-retest probability with correlations ranging from 0.61 to 0.86 [18]. Additionally, Powell et al. revealed the test’s sufficient sensitivity to uncovering global cognitive dysfunction better than test indices from the Halstead-Reitan Neuropsychological Test Battery and the Wechsler Memory Scale [19].

The standard RAVLT format begins with a list of 15 words, which an examiner reads aloud at the rate of one per second. The patient’s task is to repeat all the words he or she can remember, in any order. This procedure is carried out a total of five times. Then, the examiner presents a second list of 15 words, allowing the patient only one attempt at recall. Immediately following this, the patient is asked to remember as many words as possible from the first list. A minimum score of zero and a maximum of 15 were recorded by the psychometrician at the end of each trial depending on the number of recalled words.

The RAVLT was administered preoperatively, immediately postoperatively in the post-anesthesia care unit (PACU) except for the NSC group, at 1 week and at 3 months postoperatively. The testing was completed in a quiet area with only the subject and psychometrician present. There was only one test examiner for all patients and this examiner was trained extensively on this test prior to study commencement.

Admittedly, new learning and memory cannot be adequately analyzed using simply one summative score. However, given the nature of our surgical patient population and the timing of the testing, we feel the RAVLT and TMT offer significant insight into understanding postoperative learning and memory.

Statistical analysis

Demographic and cognitive data for the entire study population was analyzed using the Kruskal-Wallis test, one-way ANOVA, and two-way ANOVA with repeated measures. Post-hoc analyses were performed using Tukey’s honestly significant difference (HSD) or Dwass-Steel-Critchlow -Fligner test for pairwise comparisons as indicated. Bonferroni correction was applied for multiple comparisons. A p<0.05 was set for statistical significance and the analysis of variance is non-directional.

The power analysis is based on RAVLT data from the first 78 patients. The mean RAVLT delta scores (baseline to 3 months postoperatively) among the four groups (13,15,17,16 with a standard deviation of 5.0) were used to set up the power analysis. For the NSC group, we used a mean delta score of 13 that was based on the assumption that the mean RAVLT score would remain at less than 15% compared to the actual lowest delta score observed (15) in 78 patients. This is a conservative estimate since it is anticipated that an untreated nonsurgical and relatively healthy group will have lower variability in RAVLT scores. With 33 subjects per treatment group, this study will have 80% power to detect a difference in RAVLT scores among the groups. The sample size was determined using a power analysis for a one-way fixed effects analysis of variance with four levels.

## Results

In the three surgical groups, out of 99 consented subjects for this study, 76 subjects completed the trial 3 months post-surgery. Twenty-three subjects were unable to complete the study due to surgical/medical issues that resulted in protocol deviations (e.g., nasogastric tube placement, reoperation, etc.), or subjects were unwilling to continue their participation in the study.

Evaluating the four groups, patients’ mean ages were 72.8±5.5, 71.6±5.7, 73.9±6.7, and 74.8±7.7 years with no significant differences between the four groups (Table 2; p = 0.22). BMIs were 28.3±4.7, 32.9±7.8, 28.4±5.7, and 27.9±4.4, respectively. Group 1 had a statistically larger BMI (Table 2; p = 0.003).

**Table 2:** Baseline Demographics BMI: body mass index; Years Education: total years of schooling. *p = 0.003; **p = 0.027

Mean Values	Age	BMI	Years Education	Surgery Duration (minutes)	Anesthetic Duration (minutes)
Group 0 (Placebo)	72.8	28.3	12.7	135.6	191.2
Group 1 (modafinil pre, placebo post)	71.6	32.9*	12.3	164.3	217.3
Group 2 (modafinil pre, modafinil post)	73.9	28.4	14.3**	146.3	193.1
Nonsurgical controls	74.8	27.9	13.4	N/A	N/A

Educational levels measured as years in school were 12.7±0.4, 12.3±0.5, 14.2±0.5, and 13.4±0.4 for groups 0-3. The Modafinil-Modafinil group (Group 2) had a significantly higher level of education as compared to Modafinil-Placebo (Group 1), by posthoc analysis (Tukey’s HSD test, p = 0.027). Groups 0, 1, and 3 were not different from one another. The mean anesthetic duration between groups was 191.2±19.2 min, 217.3±16.8 min, and 193.1±14.4 min, respectively.

Preoperative baseline results

The RAVLT learning measure (5-trial score change) obtained preoperatively was analyzed with 2-way ANOVA with repeated measures on the 5-trial variable for a treatment effect. Main effect of treatment was significant (p = 0.030) with no significant (p = 0.203) treatment by time interaction. In other words, at baseline and before any surgical intervention, all groups showed improvement in learning (as demonstrated by an improvement in total raw score of words recalled). This effect was expected and lends validation to the RAVLT as a robust test for assessing learning.

**Figure 2:**
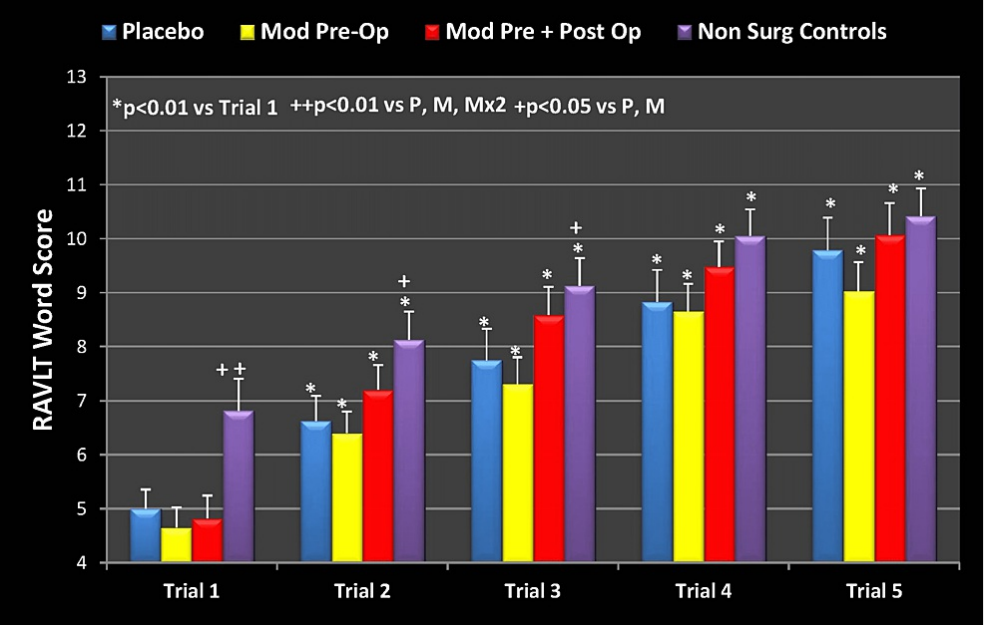
Preoperative Baseline, RAVLT Learning Measure Mod Pre-Op: modafinil preoperative; Mod Pre + Post Op: modafinil preoperative and postoperative; Non Surg Controls: nonsurgical controls; RAVLT: Rey Auditory Visual Learning Test

Incidentally, the NSC group was significantly higher in trial 1 compared to the other three groups as well as in trials 2 and 3 compared to placebo and modafinil/placebo (Group 1). This data is represented below in Figure 2.

**Figure 3:**
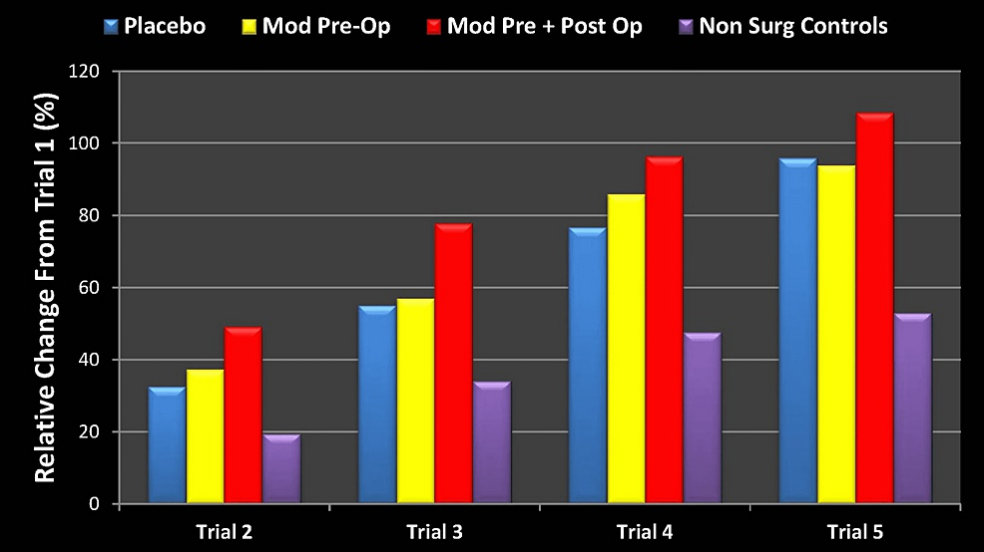
RAVLT Learning Measure Percent Change from Baseline (Trial 1) Mod Pre-Op: modafinil preoperative; Mod Pre + Post Op: modafinil preoperative and postoperative; Non Surg Controls: nonsurgical controls; RAVLT: Rey Auditory Visual Learning Test

Represented as a percent (%) change from baseline, the results are shown in Figure 3 below.

**Figure 4:**
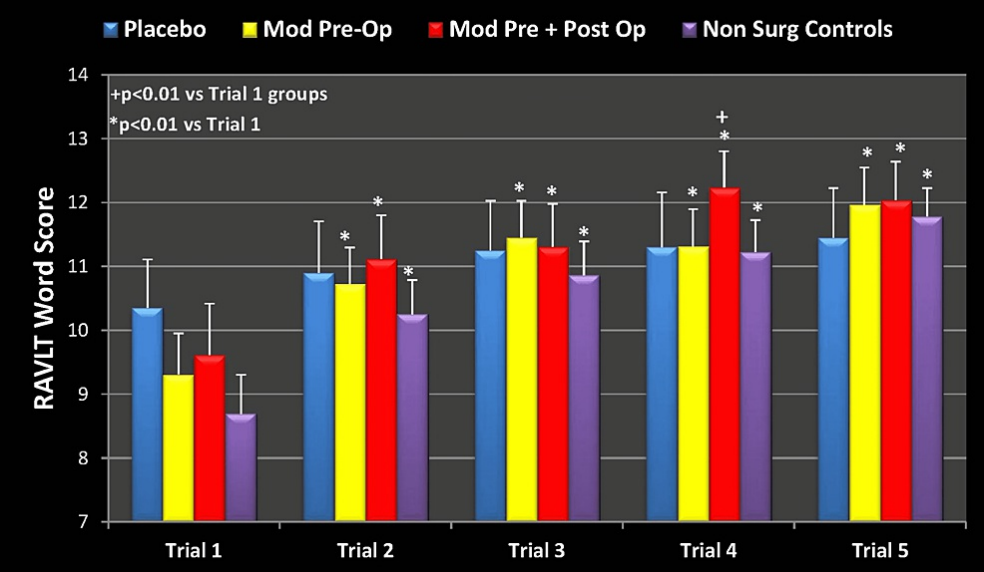
RAVLT Learning Measure (Week 1 Postoperatively) Mod Pre-Op: modafinil preoperative; Mod Pre + Post Op: modafinil preoperative and postoperative; Non Surg Controls: nonsurgical controls; RAVLT: Rey Auditory Visual Learning Test

Looking at the three groups exposed to surgery and anesthesia, we can see a flattening effect in learning in the placebo group (Figure 4). There was no statistically significant learning improvement in the RAVLT word score in this group. Importantly, this same group did have a significant improvement in learning at the preoperative baseline (Figure 2). On the other hand, there was a statistically significant improvement in learning in both groups that received modafinil (p<0.01), as well as in the NSC (p<0.01). Similar findings were seen preoperatively in Groups 1 and 2 as well as the NSCs, however, the possible detrimental cognitive effect of surgery and anesthesia did not come to light in these groups."

**Figure 5:**
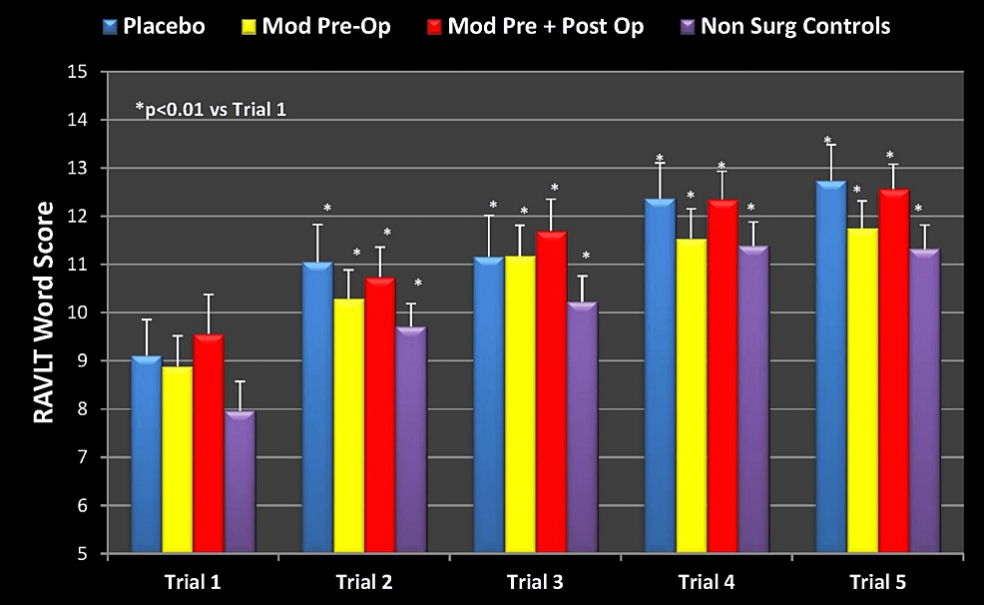
RAVLT Learning Measure ( 3 Months Postoperatively) Mod Pre-Op: modafinil preoperative; Mod Pre + Post Op: modafinil preoperative and postoperative; Non Surg Controls: nonsurgical controls; RAVLT: Rey Auditory Visual Learning Test

Figure 5 demonstrates how the subjects fared 3 months after their operation. The RAVLT learning measure (5-trial score change) obtained at 3 months postoperatively was analyzed with 2-way ANOVA with repeated measures on the 5-trial variable for a treatment effect. The learning measure was significantly (p = 0.001) increased from trial 1 within each group over the subsequent four consecutive trials. There was no significant (p = 0.397) main effect of treatment as well as no significant (p = 0.786) treatment by time interaction. The learning benefit seen in the treatment groups at 1 week was normalized at 3 months.

## Discussion

A recent review of the literature demonstrates a consensus that exposure to general anesthesia can speed cognitive decline in the elderly population [20-21]. Furthermore, there is strong evidence from animal experiments to suggest that standard doses of routine anesthetics may produce long-lasting learning and memory impairments that persist for weeks or months after anesthetic exposure [3-4,22].

Postsurgical presentation of cognitive impairment has been shown to occur as late as 3 months after surgery and significantly affects functional status [23]. Multiple etiologies have been proposed for this neurocognitive decline including age, preexisting cerebrovascular conditions, prior functioning, and ASA status, however, no definitive causative factors have been elucidated. It has been shown that POCD has long-term consequences in terms of increased all-cause mortality, premature departure from the job market due to disability, and dependency on social assistance [20,22,24]. Interestingly, a recent animal trial demonstrated that preoperative environmental enrichment was able to attenuate cognitive deficits and cytokine production in the brain [25].

In this prospective, double-blind, placebo-controlled (and controlled intervention) protocol to determine if modafinil would prevent or blunt the decline in cognitive function in the elderly undergoing noncardiac and non-neurosurgical surgery, we found that those patients who received modafinil showed significant improvement in their performance of the RAVLT, a robust test for learning, at 1 week postoperatively. However, this learning benefit had failed to be sustained at 3 months postoperatively. It is important to note that the administration of the test forecasts learning as demonstrated by the nonsurgical match group. It also can be inferred from these findings that surgery and anesthesia slow learning in the elderly and the use of modafinil may in fact inhibit this effect.

Despite the prospective nature of this study, we have identified several limitations to our results. First, we created the NSC group after the study patients were randomized. This NSC group may be considered a convenience sample, although inclusion and exclusion criteria were applied to this group. The total number of randomized subjects was 99, however, 23 subjects were unable to complete the study at 3 months. This degree of attrition could have contributed to the learning benefit seen in modafinil-treated patients. Lastly, the RAVLT learning measure test was used to assess postoperative memory and learning. Although the RAVLT test has been robustly validated in prior neuropsychiatric studies and the test was administered by the same trained examiner for all subjects, new learning and memory cannot be adequately interpreted simply using one summative score.

Modafinil is currently available as a generic formulation and only as an oral medication. The effectiveness of modafinil in reducing excessive sleepiness has been examined extensively in clinical trials for the following sleep disorders; narcolepsy, OSAHS, and SWSD. The symptoms of certain psychiatric disorders like, major depression, schizophrenia, Parkinson’s disease, and attention-deficit hyperactivity disorder may be relieved by modafinil as well [8-11].

Modafinil has wake-promoting actions similar to sympathomimetic agents, like amphetamine and methylphenidate, with only minimal adverse effects (e.g., no amphetamine-like withdrawal symptoms, although depression, anxiety, dyspnea, trembling, dizziness, vision changes, and memory loss have been reported as side effects). It has weak to negligible interactions with receptors for norepinephrine, serotonin, dopamine, gamma-aminobutyric acid (GABA), adenosine, histamine-3, melatonin, and benzodiazepines. Modafinil is not a direct- or indirect-acting dopamine receptor agonist; however, in vitro, it binds to the dopamine transporter and inhibits dopamine reuptake [26]. This activity is associated with in vivo increases in dopaminergic activity in some brain regions in animals. However, the wakefulness-promoting qualities of modafinil, unlike those of amphetamine, were not antagonized by the dopamine receptor antagonist, haloperidol, or by alpha-methyl-p-tyrosine, a dopamine synthesis inhibitor in rats [27]. Additional mechanisms of action include reductions in GABA and increases in glutamate release in various brain regions [28].

Further study should examine the dose effect, the timing of administration, and the route of administration (i.e., an intravenous formulation) to determine if the effect can be enhanced and if in fact wakefulness is improved post-surgically. Caution should be undertaken when prescribing modafinil in patients with a history of alcohol or drug abuse, a history of heart disease, depression, and mania, and in those with severe liver disease. Regular visits with the prescribing physician should be scheduled and blood pressure should be evaluated routinely.

## Conclusions

POCD is a common complication among the elderly after major surgery. A recent review of the literature demonstrates a consensus that exposure to general anesthesia can speed cognitive decline in this population. The RAVLT is a reliable and validated test with a high test-retest probability that may assess short-term auditory-verbal memory, rate of learning, presence of confabulation or confusion in memory processes, and retention of information in adults and children.

In this prospective, double-blind, placebo-controlled trial, we found that patients who received modafinil showed improvement in the RAVLT at 1 week. However, this learning benefit normalized at 3 months. Further study should examine dose effect, timing, and route of administration to determine if the effect can be enhanced and if in fact wakefulness is improved post-surgically. Additional neuropsychologic testing including the clock-drawing test, Montreal Cognitive Assessment (MoCA), or Mini-Mental State Exam (MMSE) may be pursued to strengthen our understanding of POCD in the postoperative period. Finally, laboratory testing including vitamin B12 and thyroid hormone levels and radiologic testing (e.g., computed tomography or magnetic resonance imaging) should be evaluated in further study.
